# On the Procreation of Male or Female Children, at Will

**Published:** 1859-10

**Authors:** John E. Van Molle

**Affiliations:** Brussels to the Faculty of Oglethorpe College of Savannah, for admission to the grade of Doctor in Medicine


### SELECTIONS.

*On the Procreation of Male or Female Children, at will.* Presented by John E. Van Molle, A. M., of Brussels, to the Faculty of Oglethorpe College of Savannah, for admission to the grade of Doctor in Medicine.

(From the *Oglethorpe Medical and Surgical Journal.*)

In this essay, Dr. Molle gives us the results of some experiments instituted, in the first place, as he informs us, for the advancement of science, but which have become objects of speculation in the raising of horses and cattle. Men of science, we are told, having still further extended the enquiry, endeavour to apply this philosophical fancy to other flesh besides horse-flesh and that of cattle. The following are the facts which he assures us have been obtained:

1st. That the young obtained from a mare, cow or sheep, etc., when very young, was generally a male, when the male employed was of mature age, healthy and strong.

2nd. When the female is of mature age, strong, healthy and well fed, the young is more commonly female when the male employed is young, weak or exhausted by too often repeated copulation.

3rd. That the young obtained from the same when at mature age, strong, healthy and well fed, was in nearly equal proportion, when the male employed was in a similar condition.

4th. That the young brought forth, when the female is old, were generally males when the male employed is young and strong.

5th. That the young obtained from females, when in pride, being well fed and young, were generally females, when the male was not in pride, or when ill fed, or exhausted by frequent copulation, or too old.

6th. That the young obtained from the same, when ill fed and not in pride, were generally males, when the male was well fed, young, healthy, strong and in full heat.

7th. That if the female was exhausted by labor or forced exertion, the young would be generally male, should the male employed be kept in and well fed.

8th. That the young would be female, should the female be kept at rest and the male exhausted by labor or forced exertion.

9th. To conclude, that the offspring would more generally be male or female, according to their respective physical and procreative abilities (age being taken into consideration).

From the preceding statements he derives the following deductions:

Man, being an animal, having physical and procreative faculties, analogous to those of the brutes, if a set of phenomena take place among these, the same must necessarily be produced in the human species, and if certain conditions of the physical body affect their offspring, the same physical conditions must affect the offspring in man.

Now, granting that the statements in regard to the brute creation be true, we deny that the principle involved can be extended to our species, modified as it is by civilization, and subject to so many and various influences. We therefore decline receiving these deductions, if they are based only on analogy. Is there nothing in civilization to modify the physical condition of man? Is Dr. Molle aware that some even go the length of supposing, the state of the *morale in coitu*, particularly that of the woman, has a determining influence on the organization of her offspring. Even if we do not maintain this position, to the full extent, still in the opinion of many, there is an air of probability about it sufficient to require that deductions practically ignoring and indirectly invalidating it, should be supported by an extensive collection of facts; yet when we come to examine the argument on this ground of evidence, we find but eight cases to sustain a principle, having for its whole support—a questionable analogy. Even the cases are incomplete and unsatisfactory. But Dr. Molle is not satisfied with merely establishing an analogy thus flattering between us and brutes—he favors us besides with some practical rules to guide us, when we shall have made up our minds whether we may choose to have male or female offspring. The thing is quite at our option. "We think," he proceeds to state, "we are warranted in recommending the following rules as the means to get either male or female children at will:"

To get a male child, the husband should take good substantial and somewhat stimulating food, moderate exercise, pass his time pleasantly in the gay society of women, read lascivious novels, refrain from sexual pleasures for a time previous to the procreative connections with his wife. During the same time, the wife should live sparingly, particularly on vegetables, fatigue herself every day, take some anti-aphrodisiacs and pass her time in the society of dry old women. To have female children, the opposite should be observed, the woman should live in the abundance of all good things, in the ball room, etc., but should refrain (restrain?) her passions and preserve their full force for the desired time—the male or husband, on the contrary, should reduce his physical abilities by actual labor, and at the same time, reduce his procreative propensities by frequent copious copulations.

We now learn that these two important events in man's history—his *advent* to life and union in the married state, can be best regulated, by submitting all that concerns them to the precepts of science,—that we should pair as the "beasts that perish"—that if we prefer a son to a daughter, or a daughter to a son, nothing can be more easily accomplished. We have but to follow the rules given in this essay, and the thing is done, as if by the magic wand of the enchanter, forgetting that the first of all authorities says—"It is He that hath made us, and not we ourselves." We think this essay, on grounds both physical and moral, more suited to the state of Mormon society than ours. Utah is the place of all others, where this theory of development could be tested to advantage. The old sinners, who are elders there, with their numerous wives much younger than themselves, must, according to Dr. Van Molle's "speculation," be especially blessed with female progeny—male infants, of course, must rarely be seen—the conditions for their forthcoming can scarcely exist. Thus we see how things there must be exactly dove-tailed—mutually corresponding to meet the peculiar requirements of Mormon society. Polygamy requires few males in comparison with females,—the males from exhaustion can have but few sons—hence a nice adaptation of the supply to the want. How fair and consecutive this process of reasoning appears, but unfortunately it is not founded on facts! We suspect Dr. Van Molle's theory is open to the same imputation,—it requires facts also, and until he supplies them, we will look upon his deductions as the

"baseless fabric of a vision." We are at all times willing to receive statements in connection with science, but we do object to receive suggestions falsely pretending to be based on such—suggestions that would lead us to overlook the high hopes and end of our being, and calculated to degrade and vitiate our nature.

